# Screw theory based mathematical modeling and kinematic analysis of a novel ankle rehabilitation robot with a constrained 3-PSP mechanism topology

**DOI:** 10.1007/s41315-018-0063-9

**Published:** 2018-08-07

**Authors:** Zhiwei Liao, Ligang Yao, Zongxing Lu, Jun Zhang

**Affiliations:** School of Mechanical Engineering and Automation, Fuzhou University, No. 2, Xueyuan Road, Fuzhou, 350116 Fujian China

**Keywords:** Ankle rehabilitation robot, 3-PSP parallel mechanism, Kinematics, Jacobian, Singularity, Workspace

## Abstract

As a common athletics injury in orthopedics clinic, ankle injury may affect a person’s daily life and ankle injury rehabilitation has gained increasing interests from the medical and robotic societies. A novel hybrid ankle rehabilitation robot is proposed, which composing of a serial and a parallel part. In order to analyze its kinematic performances, the parallel part of the robot is simplified as a constrained 3-PSP parallel mechanism. A mathematical model for the parallel part of the robot is established based on the screw theory. Then the inverse kinematics is obtained, and the reciprocal twists, Jacobian matrices and the singularity of the robot are analyzed. Finally the workspace of the central point on the moving platform is predicted. The kinematic analyses manifest that the proposed hybrid rehabilitation robot not only can realize three kinds of ankle rehabilitation motions, but also can eliminate singularity with enhanced workspace. The workspace of the central point reveals that the hybrid robot can fully meet the demanded rehabilitation space by comparing with the clinic demands. Our results reveals the characteristic structure of the hybrid rehabilitation robot and its superiority, it offers some basis data for the future enhancement of the device.

## Introduction

As one of the most complex joints in human skeleton, the ankle joint plays an important role in maintaining the balance of the body (Snedeker et al. 2012). Ankle injury may affect a person’s daily life. For ankle injury patients, different rehabilitation trainings are recommended from the medical perspective. Among the rehabilitation trainings, exercise therapy is one of the most commonly used approaches. Exercise therapy for ankle injury often involves motion tasks in a reasonable working space to promote the repair of damaged parts. For this purpose, some auxiliary rehabilitation devices have been developed and investigated in the past decades. According to the structural features, these auxiliary rehabilitation devices can be roughly classified into two groups: moving platform based rehabilitation device and wearable exoskeleton based rehabilitation device. For the moving platform based ankle rehabilitation device, Giorne et al. (2001) developed a six degrees-of-freedom ankle rehabilitation mechanism named ‘Rutgers’, which consists of a base platform, a moving platform and six retractable branched chains. However, investigations reveal that the workspace of this robot is limited and the control precision is affected by the drive mode of pneumatic. Besides, it is not portable. Dai et al. (2004) and Saglia et al. (2008, b) developed an ankle rehabilitation robot with 3-SPS/SP and 3-SPS/S parallel mechanism topology. This robot has two degrees-of-freedom, thus can realize plantar/dorsal flexion and inversion/eversion motions for ankle rehabilitation. In addition, the designed robot adopts actuation redundancy to eliminate singularities and to improve the dexterity. Patane and Cappa (2011) proposed an electrically actuated parallel robot with three degrees-of-freedom. This robot mainly consists of a moving platform, three fixed linear electrical actuators and three corresponding fixed length floating arms. Due to its topological configuration, the workspace of this robot is also limited. Huang et al. (2012) proposed a cable-driven ankle joint rehabilitation robot with three degrees-of-freedom, and the inverse kinematics of the mechanism has been calculated based on the closed-vector quadrilateral method. Han et al. (2015) proposed an ankle rehabilitation robot with 3-RUPS/S parallel mechanism topology and developed an inverse kinematics model for the mechanism by using the D-H method. For the wearable exoskeleton based rehabilitation device, Takemura et al. (2015) developed a Stewart-type wearable training robot for ankle and foot rehabilitation. The rehabilitation motion of ankle is realized by controlling the pressure of the six cylinders to adjust the attitude of the Stewart platform. Nevertheless, this Stewart-type rehabilitation robot suffers from complex structure, massive weight and difficult portability. Zhou et al. (2016) and Jamwal et al. (2014) proposed an adaptive wearable rehabilitation robot with three degrees-of-freedom and analyzed the kinematics, singularity and workspace of this robot. Satici et al. (2009) proposed a reconfigurable ankle rehabilitation robot based on 3-UPS and 3-RPSR parallel mechanisms. The structure of 3-UPS is adopted for exercising and strength training of ankle joints while the structure of 3-RPSR is adopted for balancing and proprioception training of ankle joints. However, the abduction/adduction of the ankle rehabilitation can’t be realized with this kind of reconfigurable mechanism. A similar wearable ankle rehabilitation robotic device was designed by Ren et al. (2016) which is suitable for early in-bed rehabilitation.

**Fig. 1:**
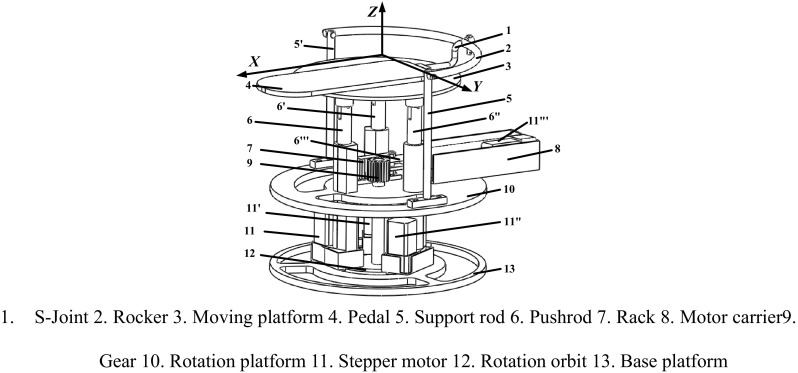
Diagram of the ankle rehabilitation robot

From the above reviews, it can be found that in the past years plenty of ankle rehabilitation robots have been proposed. Among these rehabilitation robots, most of them were designed to facilitate plantar/dorsal flexion and inversion/eversion motions. Only a few of them are capable to conduct abduction/adduction motion. Moreover, most of these ankle rehabilitation robots suffer from large size, massive weight and poor portability. In view of this, the authors propose a hybrid rehabilitation robot consisting of a serial part and a parallel part. As shown in Fig. 1, this hybrid ankle rehabilitation robot can achieve three kinds of motions pattern, i.e., plantar/dorsal flexion, inversion/eversion, abduction/adduction. The abduction/adduction motion is fulfilled by the serial part of the robot while the plantar/dorsal flexion and the inversion/eversion motions are achieved by the parallel part of the robot.

From the perspective of mechanism, the parallel part of the rehabilitation robot is a 3-PSP parallel mechanism. Literature tracking reveals that a variety 3-PSP parallel mechanism has been proposed and investigated in recent years. The studies on 3-PSP parallel mechanism mainly involve the kinematics analyses such as Jacobian matrix, singularity and kinematic sensitivity. For example, Rezaei and Akbarzadeh (2015) and Rezaei et al. (2012, 2013) analyzed the workspace, singularity and sensitivity of a 3-PSP parallel mechanism. Xie et al. (2016) developed a kinematics model for a micro 3-PSP parallel mechanism by using the closed-vector quadrilateral method. With the proposed model, the inverse and direct kinematics, the Jacobian matrices and the singularity of the mechanism were analyzed. Akbarzadeh (2015) combined a new structure of artificial neural networks (ANNs) with a 3rd order numerical algorithm and proposed an improved hybrid method for solving forward kinematics problem of 3-PSP parallel manipulators.

From the above reviews, it can be found that in the past years plenty of efforts have been carried out to analyze the kinematics of 3-PSP parallel mechanisms. Among these efforts, the constraint equations, numerical algorithm and the closed-vector quadrilateral method are the most commonly used approaches. In the present study, the authors manage to analyze the kinematics of the 3-PSP parallel mechanism from the perspective of screw theory. With the screw theory, a mathematical model for the parallel part of the hybrid ankle rehabilitation robot is established and its kinematic performances are analyzed. To be specific, the twists and reciprocal twists of each kinetic branch will be derived, followed by the constrained Jacobian matrix derivation and the constrained singularity analysis. Finally, the workspace of the parallel platform will be predicted.

## Mathematical modeling of the ankle rehabilitation mechanism

The structure of the proposed ankle rehabilitation robot is demonstrated in Fig. 1.

**Fig. 2:**
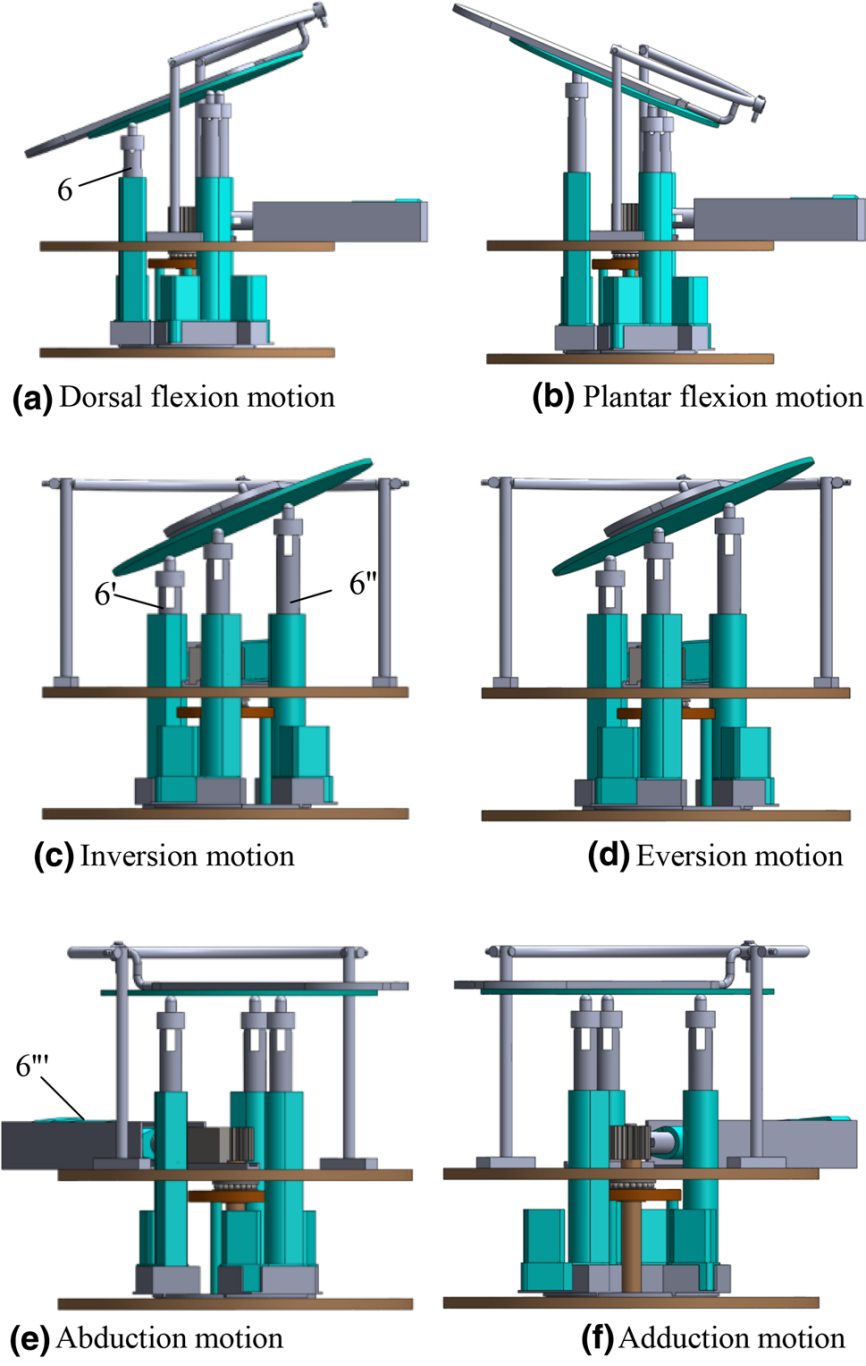
Three kinds of motions of the robot

As shown in Fig. 1, the proposed robot consists of a base platform, a rotation platform, a moving platform, a rocker, two supporting rods, three pushrods and four stepper motors. This robot can be divided into two parts, i.e., a serial part and a parallel part. The serial part of the robot consists of a rotation orbit, a rotation platform, a gear-rock pair, two support rods (5 and 5′), a rocker, an S-joint, a moving platform and a pushrod (6‴). The pushrod (6‴) is actuated by the stepper motor (11‴) to drive the rotation platform through a gear-rack pair. Thus the abduction/adduction motion of the platform along *Z* axis can be realized. The three pushrods (6, 6′ and 6″) rotate on the rotation orbit to ensure the relative position between the three pushrods and the moving platform is constant. The parallel part of the robot consists of a base platform, a moving platform, a pedal and three pushrods (6, 6′ and 6″). The pushrods (6, 6′ and 6″) are actuated by the stepper motor (11, 11′, 11″). Thus the plantar/dorsal flexion and the inversion/eversion motions of the platform along *Y* axis and *X* axis can be realized. Figure 2 illustrates the above three motion patterns of the moving platform.

From the above descriptions, one may find that the proposed hybrid robot can perform plantar/dorsal flexion, inversion/eversion and abduction/adduction motions for ankle rehabilitation. Also, this robot claims the merits of compact volume, light weight and good portability.

**Fig. 3:**
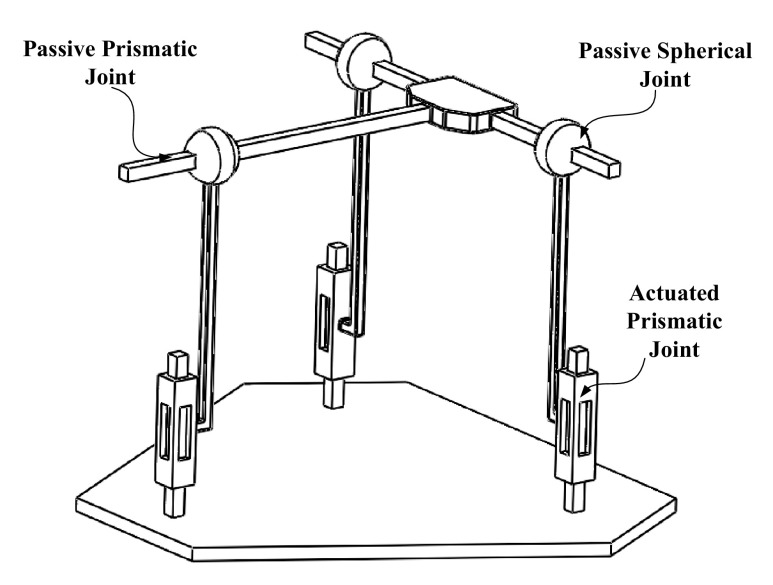
A simplified equivalent form of the constrained 3-PSP parallel mechanism

Since the structure of the serial part of the hybrid robot is comparatively simple, the following will focus on the kinematic analyses of the parallel part. As shown in Fig. 1, the three pushrods (6, 6′ and 6″) are perpendicularly contacted with the base platform, forming three higher-pairs between the interfaces. From the perspective of mechanism, the parallel part of the robot can be simplified as a 3-PSP parallel mechanism. According to the structural features, the schematic diagram of the parallel part is shown in Fig. 3.

As shown in Fig. 3, each branch chain of the constrained 3-PSP parallel mechanism consists of an actuated prismatic joint, a passive spherical joint and a passive prismatic joint. The actuated prismatic joints are perpendicular to the base platform and evenly distributed on the base platform.

## Inverse kinematics analysis

Due to the complicated topological structure of the proposed ankle rehabilitation device, the screw theory is adopted to derive more simple kinematic formulations. To facilitate the mathematical modeling, the three pushrods (6, 6′ and 6″) are represented by the letters (*A*, *B* and *C*) and the following settings are defined.

**Fig. 4:**
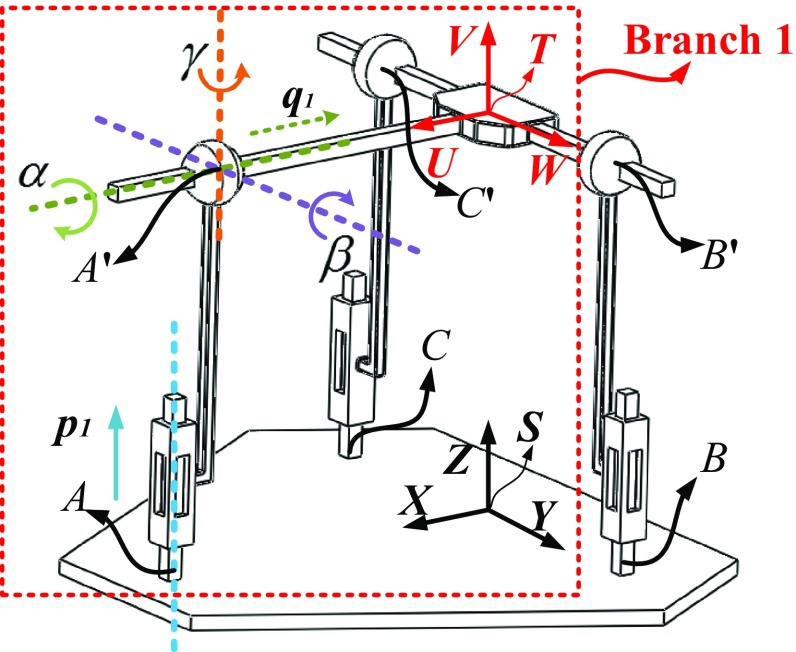
The kinematic definitions for the constraint 3-PSP parallel mechanism

As shown in Fig. 4, *A*′, *B*′ and *C*′ represent the central point of the passive spherical joint in each branch chain, respectively. A global coordinate frame *S*-*XYZ* is fixed at the midpoint between the pushrod *B* and the pushrod *C*, with *X* axis parallel to $\overrightarrow {SA}$, *Y* axis parallel to $\overrightarrow {SB}$, while *Z* axis is determined by the right-hand rule. Similarly, a tool coordinate frame *T*-*UVW* is fixed at the center of the end effector, with *U* axis parallel to $\overrightarrow {TA^\prime }$, *W* axis parallel to $\overrightarrow {TB^\prime}$, while *V* axis is determined by the right-hand rule. Taking branch 1 (*Z*-*A*-*A*′-*T*) as an example, the following definitions are made:$p_{1}$ represents the displacement of the actuated prismatic joint; $q_{1}$ represents the displacement of the passive prismatic joint; $\alpha$ represents the rotational angle along *X* axis; $\beta$ represents the rotational angle along *Y* axis.

Assuming that the distance between the end effector and the base platform is *h*, the distance between the central point of the base platform and the three pushrods (*A*, *B* and *C*) is *a*. Therefore, it can be judged from Fig. 4 that the coordinates of the pushrods (*A*, *B* and *C*) measured in the global coordinate frame *S*-*XYZ* are

1 $$ \begin{array}{*{20}l} {A = \left( {\begin{array}{*{20}l} {\frac{3a}{2}} \hfill & 0 \hfill & 0 \hfill \\ \end{array} } \right),} \hfill & {B = \left( {\begin{array}{*{20}l} 0 \hfill & {\frac{\sqrt 3 a}{2}} \hfill & 0 \hfill \\ \end{array} } \right),} \hfill & {C = \left( {\begin{array}{*{20}l} 0 \hfill & {\frac{ - \sqrt 3 a}{2}} \hfill & 0 \hfill \\ \end{array} } \right)} \hfill \\ \end{array} $$

Similarly, the coordinates of the spherical joint center (*A*′, *B*′ and *C*′) at the initial stage are

2 $$ \begin{array}{*{20}l} {A^\prime = \left( {\begin{array}{*{20}l} {\frac{3a}{2}} \hfill & 0 \hfill & h \hfill \\ \end{array} } \right),} \hfill & {B^\prime = \left( {\begin{array}{*{20}l} 0 \hfill & {\frac{\sqrt 3 a}{2}} \hfill & h \hfill \\ \end{array} } \right),} \hfill & {C^\prime = \left( {\begin{array}{*{20}l} 0 \hfill & {\frac{ - \sqrt 3 a}{2}} \hfill & h \hfill \\ \end{array} } \right)} \hfill \\ \end{array} $$

According to the screw theory (Murray et al. 1994; Gallardo-Alvarado 2005), the equation of twist is calculated as follows, Hereinafter, *i* represents the *i*th branch and j represents the *j*th joint, namely that, $\varvec{\xi}_{ij}$ represents the twist of the *j*th joint in the *i*th branch.

3 $$ \varvec{\xi}_{ij} = \left( {\begin{array}{*{20}c} { -\varvec{\omega}_{ij} \times \varvec{q}_{ij} } \\ {\varvec{\omega}_{ij} } \\ \end{array} } \right) $$

where $\varvec{\omega}_{ij} \in R^{3}$ represents a unit vector in the direction of the twist axis and $\varvec{q}_{ij} \in R^{3}$ represents any point on the axis.

For a prismatic joint, Eq. (3) can be further expressed as the following

4 $$ \varvec{\xi}_{ij} = \left( {\begin{array}{*{20}c} {\varvec{v}_{ij} } \\ {{\mathbf{0}}_{{\text{3} \times \text{1}}} } \\ \end{array} } \right) $$

where $\varvec{v}_{ij} \in R^{3}$ represents a unit vector pointing in the translational direction.

Taking branch 1 as an example, from Fig. 4 and Eqs. (3) and (4), one can derive the following twists

5 $$ \left\{ {\begin{array}{*{20}l} {\varvec{\xi}_{{\text{11}}} = \left( {\begin{array}{*{20}l} 0 \hfill & 0 \hfill & 1 \hfill & 0 \hfill & 0 \hfill & 0 \hfill \\ \end{array} } \right)^{\text{T}} } \hfill \\ {\varvec{\xi}_{{\text{12}}} = \left( {\begin{array}{*{20}l} 0 \hfill & h \hfill & 0 \hfill & 1 \hfill & 0 \hfill & 0 \hfill \\ \end{array} } \right)^{\text{T}} } \hfill \\ {\varvec{\xi}_{{\text{13}}} = \left( {\begin{array}{*{20}l} { - h} \hfill & 0 \hfill & {3a/2} \hfill & 0 \hfill & 1 \hfill & 0 \hfill \\ \end{array} } \right)^{\text{T}} } \hfill \\ {\varvec{\xi}_{{\text{14}}} = \left( {\begin{array}{*{20}l} 0 \hfill & { - 3a/2} \hfill & 0 \hfill & 0 \hfill & 0 \hfill & 1 \hfill \\ \end{array} } \right)^{\text{T}} } \hfill \\ {\varvec{\xi}_{{\text{15}}} = \left( {\begin{array}{*{20}l} 1 \hfill & 0 \hfill & 0 \hfill & 0 \hfill & 0 \hfill & 0 \hfill \\ \end{array} } \right)^{\text{T}} } \hfill \\ \end{array} } \right. $$

If we define $\varvec{g}_{{l_{j - 1} l_{j} }} \left( {\theta_{ij} } \right)$ as the transformation between the adjacent joints, the overall transformation matrix is

6 $$ \varvec{g}_{st} \left( {\varvec{\theta}_{ij} } \right) = \varvec{g}_{{sl_{\text{1}} }} \left( {\theta_{{i\text{1}}} } \right)\varvec{g}_{{l_{\text{1}} l_{\text{2}} }} \left( {\theta_{{i\text{2}}} } \right)\varvec{g}_{{l_{\text{2}} l_{\text{3}} }} \left( {\theta_{{i\text{3}}} } \right) \ldots \varvec{g}_{{l_{n - 1} l_{n} }} \left( {\theta_{in} } \right)\varvec{g}_{{l_{n} t}} $$

The motion of individual joints can be obtained as

7 $$ \varvec{g}_{ab} \left( {\varvec{\theta}_{ij} } \right) = \varvec{e}^{{\widehat{\varvec{\xi}}_{ij} \theta_{ij} }} \varvec{g}_{ab} \left( \text{0} \right) $$

where $\varvec{e}^{{\widehat{\varvec{\xi}}_{ij} \theta_{ij} }}$ represents the exponential of twists and $\varvec{\theta}_{ij} \text{ = (}\theta_{i1} ,\theta_{i2} ,\theta_{i3} , \ldots ,\theta_{in} \text{)}$ represents a set of joint angles. And in this paper, we hypothesize the pitch of the twist is 0.

8 $$ \varvec{e}^{{\widehat{\varvec{\xi}}_{ij} \theta_{ij} }} = \left[ {\begin{array}{*{20}c} {\varvec{e}^{{\hat{\varvec{\omega }}_{ij} \theta_{ij} }} } & {\left( {{\mathbf{I}} - \varvec{e}^{{\hat{\varvec{\omega }}_{ij} \theta_{ij} }} } \right)\varvec{q}} \\ {{\mathbf{0}}_{{\text{1} \times \text{3}}} } & \text{1} \\ \end{array} } \right] $$

where $\varvec{e}^{{\hat{\varvec{\omega }}_{ij} \theta_{ij} }} = {\mathbf{I}} + \hat{\varvec{\omega }}_{ij} \text{sin}\left( {\theta_{ij} } \right) + \hat{\varvec{\omega }}_{ij}^{2} \left( {\text{1} - \text{cos}\theta_{ij} } \right)$. And we have

9 $$ \varvec{e}^{{\widehat{\varvec{\xi}}_{11} \theta_{11} }} = \left[ {\begin{array}{*{20}c} 1 & 0 & 0 & 0 \\ 0 & 1 & 0 & 0 \\ 0 & 0 & 1 & {p_{1} } \\ 0 & 0 & 0 & 1 \\ \end{array} } \right] $$

10 $$ \varvec{e}^{{\widehat{\varvec{\xi}}_{12} \theta_{12} }} = \left[ {\begin{array}{*{20}c} 1 & 0 & 0 & 0 \\ 0 & {\cos \alpha } & { - \sin \alpha } & {h\sin \alpha } \\ 0 & {\sin \alpha } & {\cos \alpha } & {h(1 - \cos \alpha )} \\ 0 & 0 & 0 & 1 \\ \end{array} } \right] $$

11 $$ \varvec{e}^{{\widehat{\varvec{\xi}}_{13} \theta_{13} }} = \left[ {\begin{array}{*{20}c} {\cos \beta } & 0 & {\sin \beta } & {a - a\cos \beta - h\sin \beta } \\ 0 & 1 & 0 & 0 \\ { - \sin \beta } & 0 & {\cos \beta } & {a\sin \beta + h(1 - \cos \beta )} \\ 0 & 0 & 0 & 1 \\ \end{array} } \right] $$

12 $$ \varvec{e}^{{\widehat{\varvec{\xi}}_{14} \theta_{14} }} = \left[ {\begin{array}{*{20}c} 1 & 0 & 0 & 0 \\ 0 & 1 & 0 & 0 \\ 0 & 0 & 1 & 0 \\ 0 & 0 & 0 & 1 \\ \end{array} } \right] $$

13 $$ \varvec{e}^{{\widehat{\varvec{\xi}}_{15} \theta_{15} }} = \left[ {\begin{array}{*{20}c} 1 & 0 & 0 & { - q_{1} } \\ 0 & 1 & 0 & 0 \\ 0 & 0 & 1 & 0 \\ 0 & 0 & 0 & 1 \\ \end{array} } \right] $$

The configuration of the end effector at the initial stage $\varvec{g}_{st} \left( \text{0} \right)$ is

14 $$ \varvec{g}_{st} \left( \text{0} \right) = \left[ {\begin{array}{*{20}c} 1 & 0 & 0 & 0 \\ 0 & 1 & 0 & 0 \\ 0 & 0 & 1 & h \\ 0 & 0 & 0 & 1 \\ \end{array} } \right] $$

According to Eq. (7), we have

15 $$ \varvec{g}_{st} \left( {\varvec{\theta}_{{\text{1}j}} } \right) = \varvec{e}^{{\widehat{\varvec{\xi}}_{11} \theta_{11} }} \varvec{e}^{{\widehat{\varvec{\xi}}_{12} \theta_{12} }} \varvec{e}^{{\widehat{\varvec{\xi}}_{13} \theta_{13} }} \varvec{e}^{{\widehat{\varvec{\xi}}_{14} \theta_{14} }} \varvec{e}^{{\widehat{\varvec{\xi}}_{15} \theta_{15} }} \varvec{g}_{st} \left( \text{0} \right) $$

The inverse position of branch 1 can be expressed as follows

16 $$ \varvec{g}_{st} \left( {\varvec{\theta}_{{\text{1}j}} } \right) = \left[ {\begin{array}{*{20}c} {\cos \beta } & 0 & {\sin \beta } & { - q_{1} \cos \beta - \frac{3a(\cos \beta - 1)}{2}} \\ {\sin \alpha \sin \beta } & {\cos \alpha } & { - \cos \beta \sin \alpha } & { - \sin \alpha \sin \beta (q_{1} + \frac{3a}{2})} \\ { - \cos \alpha \sin \beta } & {\sin \alpha } & {\cos \alpha \cos \beta } & {p_{1} + h + q_{1} \cos \alpha \sin \beta + \frac{3a\cos \alpha \sin \beta }{2}} \\ 0 & 0 & 0 & 1 \\ \end{array} } \right] $$

With the same derivation process, the inverse positions of branch 2 and 3 can be obtained as follows

17 $$ \varvec{g}_{st} \left( {\varvec{\theta}_{{\text{2}j}} } \right) = \left[ {\begin{array}{*{20}c} {\cos \beta } & 0 & {\sin \beta } & 0 \\ {\sin \alpha \sin \beta } & {\cos \alpha } & { - \cos \beta \sin \alpha } & {\frac{\sqrt 3 a}{2} - q_{2} \cos \alpha - \frac{\sqrt 3 a\cos \alpha }{2}} \\ { - \cos \alpha \sin \beta } & {\sin \alpha } & {\cos \alpha \cos \beta } & {p_{2} + h - q_{2} \sin \alpha + \frac{\sqrt 3 a\sin \alpha }{2}} \\ 0 & 0 & 0 & 1 \\ \end{array} } \right] $$

18 $$ \varvec{g}_{st} \left( {\varvec{\theta}_{{\text{3}j}} } \right) = \left[ {\begin{array}{*{20}c} {\cos \beta } & 0 & {\sin \beta } & 0 \\ {\sin \alpha \sin \beta } & {\cos \alpha } & { - \cos \beta \sin \alpha } & {\frac{ - \sqrt 3 a}{2} + q_{3} \cos \alpha + \frac{\sqrt 3 a\cos \alpha }{2}} \\ { - \cos \alpha \sin \beta } & {\sin \alpha } & {\cos \alpha \cos \beta } & {p_{3} + h + q_{3} \sin \alpha + \frac{\sqrt 3 a\sin \alpha }{2}} \\ 0 & 0 & 0 & 1 \\ \end{array} } \right] $$

where $p_{2}$ and $p_{3}$ represent the displacements of the actuated prismatic joints while $q_{2}$ and $q_{3}$ represent the displacements of the passive prismatic joints.

With Eqs. (16), (17) and (18), the displacements of the pushrods measured in *S*-*XYZ* are

19 $$ \left\{ {\begin{array}{*{20}l} {p_{1} = - \frac{3a\sin \beta }{2\cos \alpha cos\beta }} \hfill \\ {p_{2} = \frac{\sqrt 3 a\sin \alpha \cos \beta }{2\cos \alpha cos\beta }} \hfill \\ {p_{3} = - \frac{\sqrt 3 a\sin \alpha \cos \beta }{2\cos \alpha cos\beta }} \hfill \\ \end{array} } \right. $$

According to Eq. (19), the displacement of each pushrod depends on the selection of the global coordinate frame *S*-*XYZ*, if the coordinates of each pushrod related to the *S*-*XYZ* can be expressed as follows

20 $$ A\text{ = }\left( {\begin{array}{*{20}c} {x_{a} } & {\text{y}_{a} } & \text{0} \\ \end{array} } \right)\text{, }B\text{ = }\left( {\begin{array}{*{20}c} {x_{b} } & {\text{y}_{b} } & \text{0} \\ \end{array} } \right)\text{,}C\text{ = }\left( {\begin{array}{*{20}c} {x_{c} } & {x_{c} } & \text{0} \\ \end{array} } \right) $$

The inverse kinematics of the parallel part of the novel ankle rehabilitation robot can be obtained as follows

21 $$ p_{i} = \frac{{y_{i} \sin \alpha \cos \beta - x_{i} \sin \beta }}{\cos \alpha \cos \beta } $$

## Reciprocal twists, Jacobian, and singularity analysis

Based on the above discussion, the twists of branch 1 are calculated in Eq. (5). Similarly, the twists of branch 1 and 2 can be derived as follows

22 $$ \left\{ {\begin{array}{*{20}l} {\varvec{\xi}_{21} = \left( {\begin{array}{*{20}l} 0 \hfill & 0 \hfill & 1 \hfill & 0 \hfill & 0 \hfill & 0 \hfill \\ \end{array} } \right)^{\text{T}} } \hfill \\ {\varvec{\xi}_{22} = \left( {\begin{array}{*{20}l} 0 \hfill & h \hfill & { - \sqrt 3 a/2} \hfill & 1 \hfill & 0 \hfill & 0 \hfill \\ \end{array} } \right)^{\text{T}} } \hfill \\ {\varvec{\xi}_{23} = \left( {\begin{array}{*{20}l} { - h} \hfill & 0 \hfill & 0 \hfill & 0 \hfill & 1 \hfill & 0 \hfill \\ \end{array} } \right)^{\text{T}} } \hfill \\ {\varvec{\xi}_{24} = \left( {\begin{array}{*{20}l} {\sqrt 3 a/2} \hfill & 0 \hfill & 0 \hfill & 0 \hfill & 0 \hfill & 1 \hfill \\ \end{array} } \right)^{\text{T}} } \hfill \\ {\varvec{\xi}_{25} = \left( {\begin{array}{*{20}l} 0 \hfill & { - 1} \hfill & 0 \hfill & 0 \hfill & 0 \hfill & 0 \hfill \\ \end{array} } \right)^{\text{T}} } \hfill \\ {\varvec{\xi}_{31} = \left( {\begin{array}{*{20}l} 0 \hfill & 0 \hfill & 1 \hfill & 0 \hfill & 0 \hfill & 0 \hfill \\ \end{array} } \right)^{\text{T}} } \hfill \\ {\varvec{\xi}_{32} = \left( {\begin{array}{*{20}l} 0 \hfill & h \hfill & {\sqrt 3 a/2} \hfill & 1 \hfill & 0 \hfill & 0 \hfill \\ \end{array} } \right)^{\text{T}} } \hfill \\ {\varvec{\xi}_{33} = \left( {\begin{array}{*{20}l} { - h} \hfill & 0 \hfill & 0 \hfill & 0 \hfill & 1 \hfill & 0 \hfill \\ \end{array} } \right)^{\text{T}} } \hfill \\ {\varvec{\xi}_{34} = \left( {\begin{array}{*{20}l} { - \sqrt 3 a/2} \hfill & 0 \hfill & 0 \hfill & 0 \hfill & 0 \hfill & 1 \hfill \\ \end{array} } \right)^{\text{T}} } \hfill \\ {\varvec{\xi}_{35} = \left( {\begin{array}{*{20}l} 0 \hfill & 1 \hfill & 0 \hfill & 0 \hfill & 0 \hfill & 0 \hfill \\ \end{array} } \right)^{\text{T}} } \hfill \\ \end{array} } \right. $$

The reciprocal twists are the force constraint of the rigid body in the process of the motions, and it is also the structural constraints of the mechanism (Zhang et al. 2015; Cai et al. 2014). Assume that $\varvec{\xi}_{i}^{c}$ is the reciprocal twist of the twists $\varvec{\xi  }_{ij}$, namely $\varvec{\xi}_{i}^{c}$ is a reciprocal twist of branch *i*, and we have

23 $$ \varvec{\xi}_{ij} \odot\varvec{\xi}_{i}^{\varvec{c}} = 0 $$

Hence, the reciprocal twists of the parallel part of the robot can be expressed as

24 $$ \left\{ {\begin{array}{*{20}l} {\varvec{\xi}_{\text{1}}^{c} = \left( {\begin{array}{*{20}l} { - h} \hfill & 0 \hfill & {3a/2} \hfill & 0 \hfill & 1 \hfill & 0 \hfill \\ \end{array} } \right)^{\text{T}} } \hfill \\ {\varvec{\xi}_{\text{2}}^{c} = \left( {\begin{array}{*{20}l} 0 \hfill & h \hfill & { - 3a/2} \hfill & 1 \hfill & 0 \hfill & 0 \hfill \\ \end{array} } \right)^{\text{T}} } \hfill \\ {\varvec{\xi}_{\text{3}}^{c} = \left( {\begin{array}{*{20}l} 0 \hfill & h \hfill & {3a/2} \hfill & 1 \hfill & 0 \hfill & 0 \hfill \\ \end{array} } \right)^{\text{T}} } \hfill \\ \end{array} } \right. $$

According to the above reciprocal twists, we can find that the directions of the constraint forces are parallel to the *X*–*Y* plane. In other words, the robot is restricted by three constraint forces perpendicular to *Z* axis.

The constrained Jacobian matrix $\varvec{J}_{c}$ is given.

25 $$ \varvec{J}_{c} = \left[ {\begin{array}{*{20}c} {\varvec{\xi}_{\text{1}}^{c} } & {\varvec{\xi}_{\text{2}}^{c} } & {\varvec{\xi}_{\text{2}}^{c} } \\ \end{array} } \right] = \left[ {\begin{array}{*{20}l} { - h} \hfill & 0 \hfill & 0 \hfill \\ 0 \hfill & h \hfill & h \hfill \\ {\frac{3a}{2}} \hfill & { - \frac{3a}{2}} \hfill & {\frac{3a}{2}} \hfill \\ 0 \hfill & 1 \hfill & 1 \hfill \\ 1 \hfill & 0 \hfill & 0 \hfill \\ 0 \hfill & 0 \hfill & 0 \hfill \\ \end{array} } \right] $$

From the above equation, it can be found that there exists no constrained singularity of the parallel mechanism because rank (*J*_*c*_) = 3.

With the screw theory, the kinematic Jacobian matrix of a kinematic chain can be written as

26 $$ \varvec{J}_{ST}^{k} \left( {\varvec{\theta}_{ij} } \right) = \left[ {\begin{array}{*{20}l} {\varvec{\xi}_{i1} ^\prime } \hfill & \ldots \hfill & {\varvec{\xi}_{in} ^\prime } \hfill \\ \end{array} } \right] $$

where $$ \xi_{ij} ^\prime = {\text{Ad}}_{{\left( {\begin{array}{*{20}c} {e^{{\xi_{i1} \theta_{i1} }} } & {. \ldots } & {e^{{\xi_{ij - 1} \theta_{ij - 1} }} } \\ \end{array} } \right)}} \xi_{ij} $$, ${\mathbf{Ad}}_{\varvec{g}}$ is a 6 × 6 adjoint transformation matrix associated with *g*.

27 $$ {\mathbf{Ad}}_{\varvec{g}} = \left[ {\begin{array}{*{20}c} \varvec{R} & {\hat{\varvec{P}}\varvec{R}} \\ {{\mathbf{0}}_{{\text{3} \times \text{3}}} } & \varvec{R} \\ \end{array} } \right] $$

For example, the kinematic Jacobian matrix of branch 1 can be expressed as follows.

28 $$ \varvec{J}_{{\text{ST}}}^{k} \left( {\varvec{\theta}_{{\text{1}j}} } \right) = \left[ {\begin{array}{*{20}l} {\varvec{\xi}_{{\text{11}}} ^\prime } \hfill & {\varvec{\xi}_{{\text{12}}} ^\prime } \hfill & {\varvec{\xi}_{{\text{13}}} ^\prime } \hfill & {\varvec{\xi}_{{\text{14}}} ^\prime } \hfill & {\varvec{\xi}_{{\text{15}}} ^\prime } \hfill \\ \end{array} } \right] $$

namely,

29 $$ \varvec{J}_{{\text{ST}}}^{k} \left({\varvec{\theta}_{{\text{1}j}} } \right) = \left[{\begin{array}{*{20}l} 0 \hfill & \quad  0 \hfill & \quad  { - \left( {h +p_{1} } \right)\cos \alpha } \hfill & \quad  {\left( {h + p_{ 1} }\right)\sin \alpha \cos \beta } \hfill & \quad  { - \cos \beta } \hfill\\ 0 \hfill & \quad  {h + p_{1} } \hfill & \quad  { - \frac{3a}{2}\sin\alpha } \hfill & \quad  {(p_{1} + h)\sin \beta - \frac{3a}{2}\cos\alpha \cos \beta } \hfill & \quad  { - \sin \alpha \sin \beta } \hfill\\ 1 \hfill & \quad  0 \hfill & \quad  {\frac{3a}{2}\sin \alpha } \hfill& \quad  { - \frac{3a}{2}\cos \alpha \cos \beta } \hfill & \quad  {\cos\alpha \sin \beta } \hfill \\ 0 \hfill & \quad  1 \hfill & \quad  0 \hfill& \quad  {\sin \beta } \hfill & \quad  0 \hfill \\ 0 \hfill & \quad  0 \hfill& \quad  {\cos \alpha } \hfill & \quad  { - \sin \alpha \cos \beta }\hfill & \quad  0 \hfill \\ 0 \hfill & \quad  0 \hfill & \quad  {\sin \alpha} \hfill & \quad  {\cos \alpha \cos \beta } \hfill & \quad  0 \hfill \\\end{array} } \right] $$

If $\beta \text{ = }\frac{\uppi}{2}$, by analyzing Eq. (29), $\varvec{J}_{{\text{ST}}}^{k} \left( {\varvec{\theta}_{{\text{1}j}} } \right)$ can be simplified as follows

30 $$ \varvec{J}_{{\text{ST}}}^{k} \left({\varvec{\theta}_{{\text{1}j}} } \right) = \left[{\begin{array}{*{20}l} 0 \hfill & \quad  0 \hfill & \quad  { - \left( {h +p_{1} } \right)\cos \alpha } \hfill & \quad  0 \hfill & \quad  0 \hfill \\0 \hfill & \quad  {h + p_{1} } \hfill & \quad  { - \frac{3a}{2}\sin \alpha} \hfill & \quad  {p_{1} + h} \hfill & \quad  { - \sin \alpha } \hfill \\1 \hfill & \quad  0 \hfill & \quad  {\frac{3a}{2}\sin \alpha } \hfill& \quad  0 \hfill & \quad  {\cos \alpha } \hfill \\ 0 \hfill & \quad  1\hfill & \quad  0 \hfill & \quad  1 \hfill & \quad  0 \hfill \\ 0 \hfill& \quad  0 \hfill & \quad  {\cos \alpha } \hfill & \quad  0 \hfill & \quad  0\hfill \\ 0 \hfill & \quad  0 \hfill & \quad  {\sin \alpha } \hfill & \quad0 \hfill & \quad  0 \hfill \\ \end{array} } \right] $$

From the above equation, it can be found that there exists kinematic singularity of the parallel mechanism if $\beta = \frac{\uppi}{2}$, because $rank\left( {\varvec{J}_{{\text{ST}}}^{k} \left( {\varvec{\theta}_{{\text{1}j}} } \right)} \right) = 4$. However, when $\beta  = \frac{\uppi}{2}$, the displacements of the pushrods (6, 6′, 6″) achieve *p*_1_ → ∞, *p*_2_/*p*_3_ → − ∞ or *p*_1_ → − ∞, *p*_2_/*p*_3_ → ∞. Because of the finite length of the pushrods, there does not exist kinematic singularity in each branch. Since the kinematic Jacobian matrix of branch 2 and branch 3 are in the same form, there does not exist kinematic singularity in the whole parallel mechanism of the robot.

## Workspace analysis

In this section, the workspace of the moving platform is predicted. The parameters of the robot mainly include the length of pushrods *L*, the radius of moving platform *R*, the distance between the end effector and the base platform *h* and the distance between the center point of the base platform and the three pushrods (6, 6′ and 6″) *a*.

In the present study, *L* = 150 mm, *R* = 150 mm, *h* = 165 mm and *a* = 50 mm. Because when robot only performs an inversion/eversion motion, the angle range of the motion only related to the displacements of the pushrod (6′) *p*_2_ and the pushrod (6″) *p*_3_. Moreover, *p*_2_ = − *p*_3_. In order to obtain the maximum range of the motion, the extension of the pushrods is set to be 75 mm at the initial stage, the displacements of the actuated prismatic joints $p_{\text{1}} /p_{\text{2}} /p_{\text{3}}$ and the displacements of the passive prismatic joints $q_{1} /q_{2} /q_{3}$ are set as

31 $$ \begin{aligned} - 75\,\text{mm} \le p_{1} \le 75\,\text{mm},\quad - 75\,\text{mm} \le q_{1} \le 100\,\text{mm} \hfill \\ - 75\,\text{mm} \le p_{2} \le 75\,\text{mm},\quad - 4 3. 3\,\text{mm} \le q_{2} \le 86.6\,\text{mm} \hfill \\ - 75\,\text{mm} \le p_{3} \le 75\,\text{mm},\quad - 86.6\,\text{mm} \le q_{3} \le 4 3. 3\,\text{mm} \hfill \\ \end{aligned} $$

With Eqs. (19) and (30), the angle range of the inversion/eversion motion is obtained as follows

32 $$ - 60^\circ \le \alpha \le 60^\circ $$

**Fig. 5:**
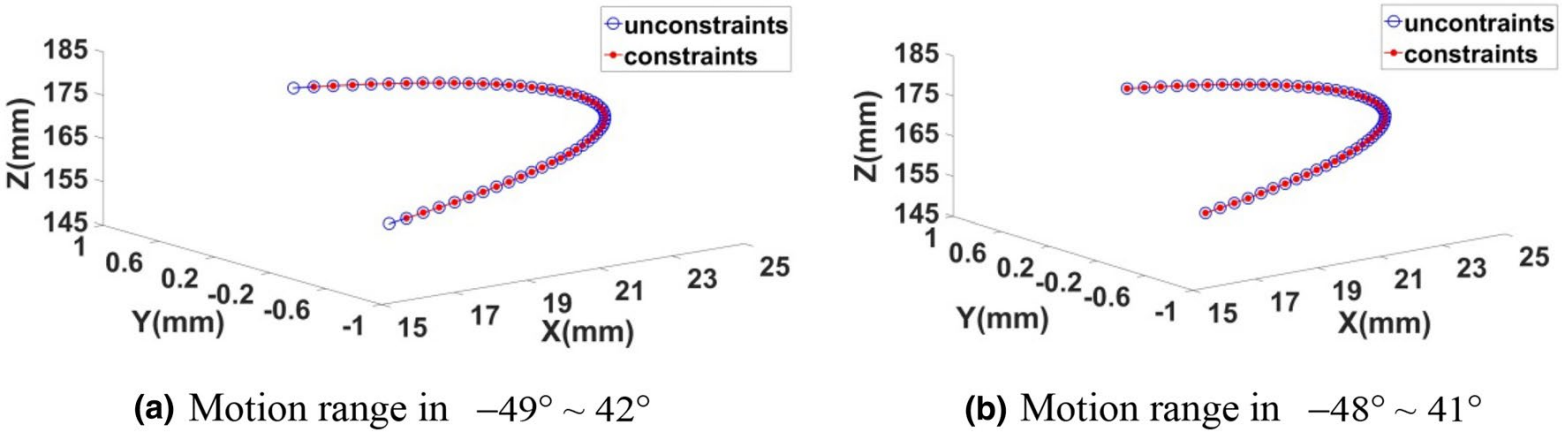
Trajectory of plantar/dorsal flexion motion with two different ranges

In order to predict the angle range of the plantar/dorsal flexion motion, we estimate the range of the moving platform and set the ranges of the plantar/dorsal flexion as (−49°, 42°) and (−48°, 41°) respectively. Then we analyze the trajectory of these two motion patterns with constraints and without constraints of the structure of the mechanism. The results are shown in Fig. 5

As shown in Fig. 5, the trajectory of plantar/dorsal flexion motion with two different ranges are proposed, if we set the motion range as (−49° ~ 42°), the robot cannot cover the whole motion; but if we set the motion range as (−48° ~ 41°), the whole motion can be covered by the robot. From the above descriptions, we can draw a conclusion that, the maximum angle of the dorsal flexion can be set as 41°, and the maximum angle of the plantar flexion can be set as 48°. So the angle range of the plantar/dorsal flexion motion can be set as

33 $$ - 48^\circ \le \beta \le 41^\circ $$

According to Wang et al. (2013), Aman et al. (2015), Roaas and Andersson (1982), Chen et al. (2017) and Zhao et al. (2014), the security angles for ankle rehabilitation exercise are listed in Table 1.

**Table 1:** Ranges of angle for ankle motion

Type of motion	Max. allowable motion	Motion range of robot
Plantar flexion	37°–45°	0°–48°
Dorsal flexion	20°–30°	0°–41°
Inversion	14.5°–22°	0°–60°
Eversion	10°–20°	0°–60°

**Fig. 6:**
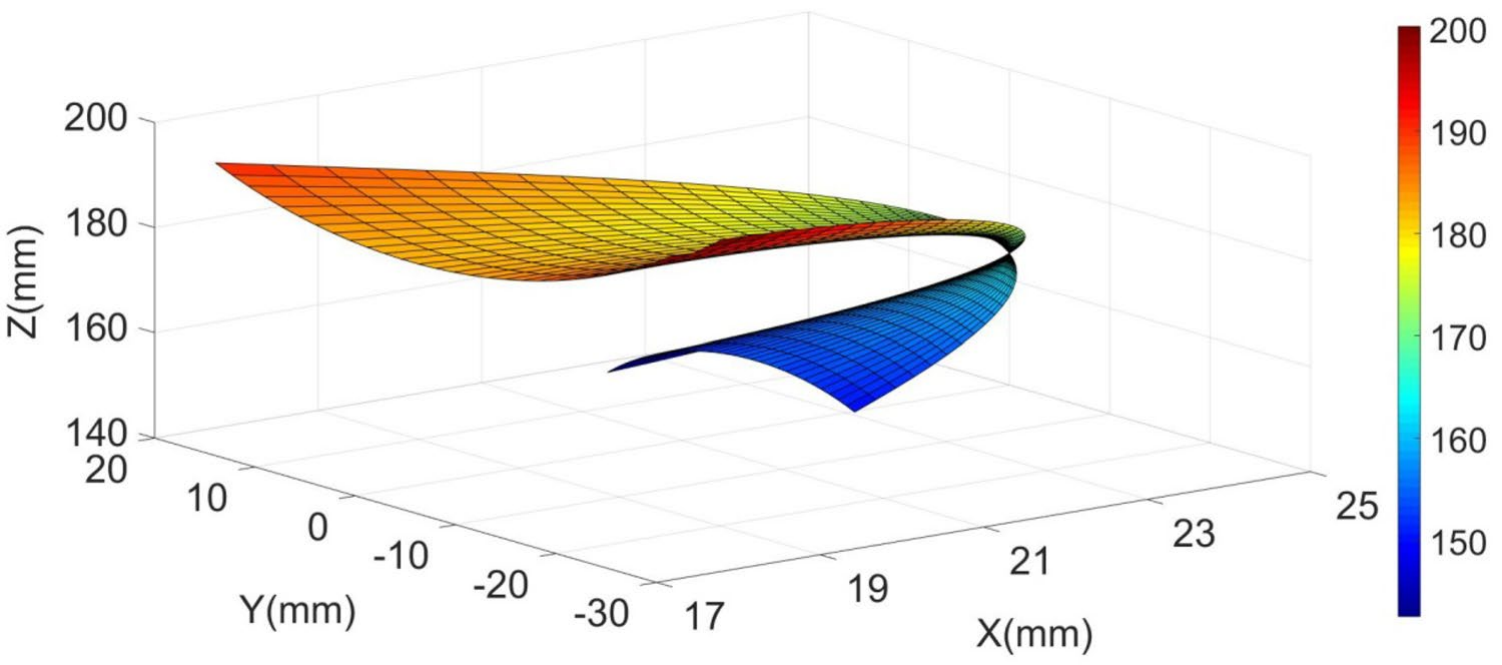
Workspace of the central point on the moving platform

According to the angle range shown in Table 1, the robot proposed in this paper can fully meet the angle demands of ankle rehabilitation. Noticing safety is one of the most important factors in rehabilitation, we dedicatedly control the motion of platform to ensure the range of the rehabilitation motion does not exceed the clinical safety range. The details of the determination of rehabilitation motion, trajectory planning and motor control can be referred to our previous work (Liao et al. 2016, 2017, 2018; Yao et al. 2018). On this basis, the plantar/dorsal flexion (along *Y* axis) and inversion/eversion (along *X* axis) are set as *β* ∊ (− 45°, 30°) and *α* ∊ (− 22°, 20°). The workspace of the central point on the moving platform under the combined motion of plantar/dorsal flexion and inversion/eversion is depicted in Fig. 6.

As shown in Fig. 6, when the platform performs a combined motion of plantar/dorsal flexion and inversion/eversion, the workspace of the central point is divided into two parts by the initial point. The upper part of the initial point is the combined motion of plantar flexion and inversion/eversion; similarly, the lower part of the initial point is the combined motion of dorsal flexion and inversion/eversion. Further investigation reveals that the motion with constraints along *X* axis, *Y* axis and *Z* axis are located in the intervals of 17.7–25.0 mm, −21.9 to 18.9 and 141.9–200 mm, respectively. In summary, the robot proposed can fully meet the demands of ankle rehabilitation exercise. The above work offers some basis data for the future enhancement of the ankle rehabilitation device.

## Conclusion

In this paper, a novel hybrid ankle rehabilitation robot is proposed, which is composed of a serial part and a parallel part. The proposed hybrid robot can achieve three motion patterns, i.e., plantar/dorsal flexion, inversion/eversion, abduction/adduction. The structure of the hybrid robot is analyzed and the parallel part of the robot is simplified as a 3-PSP parallel mechanism. A mathematical model is established for the 3-PSP parallel mechanism. On this basis, an inverse kinematic analysis is carried out, and the Jacobian matrices and singularity of the parallel mechanism are deduced, the results reveal that there exists no constrained and kinematic singularity of the robot. The workspace of the robot is predicted. The comparison reveals that the hybrid robot can fully meet the demanded rehabilitation space.

The present study provides some basis for further investigations such as trajectory planning, rehabilitation strategies and so on.
